# The Sagittal Pelvic Thickness: A Determining Parameter for the Regulation of the Sagittal Spinopelvic Balance

**DOI:** 10.5402/2013/364068

**Published:** 2013-07-24

**Authors:** Legaye Jean

**Affiliations:** Department of Orthopaedic Surgery, University Hospital UCL Mont-Godinne, 5530 Yvoir, Belgium

## Abstract

*Objective.* To propose and validate a dimensional parameter, the sagittal pelvic thickness (SPT) (distance between the middle point of the upper sacral plate and the femoral heads axis, expressed as a ratio with the length of the upper plate of S1: (SPT/S1) for the analysis of the sagittal balance of the pelvispinal unit. *Methods.* The parameters were analysed on standing radiographic imaging and compared for normal, low back pain, children, and spondylolysis cases. *Results.* Values of SPT/S1 were observed significantly higher in high grade spondylolysis populations and in children (3,5 and 3,7) than in normal population (3,3). A geometrical connection with the classical angular parameters validated SPT/S1. *Conclusion.* SPT/S1 was considered reflecting the lever arm of action of spinopelvic muscles and ligaments and describing the ability of a subject to compensate a sagittal unbalance. It was proposed as an anatomical and functional pelvic parameter.

## 1. Introduction

A strict relation was described between the sagittal pelvic anatomy and the sagittal shape of the spine, particularly the amount of lordosis needed for each individual. Therefore, angular parameters were recommended because they are usable disregarding the size of the subjects [1–5].

In the same way, the distinction was established a long time ago by morphologists and paleontologists between the “pelvis in tension” of the quadrupeds and the “pelvis in pressure” characterizing the bipedalism [6]. They were distinguished according to their more or less lengthened form, defined by the distance between the upper sacral plate and the coxofemoral joints: the sagittal pelvic thickness (SPT). In spite of characterizing the sagittal pelvic anatomy as well as, angular parameters, SPT was poorly studied. By a radiographic study, we investigated here its significance on the spinopelvic sagittal balance and its clinical relevance.

## 2. Material and Methods

Angular and dimensional parameters were measured on 272 lateral radiographies including the pelvis, the femoral heads and the lumbar column, in standardized standing position [7]. For each, a scaling was incorporated allowing correction of the radiographic distortion. Data of four population groups were analyzed (Table 1). 

The first group comprised 61 healthy voluntaries (column A). Data were obtained several years ago from for original orthopaedic studies [5]. At this time, these subjects provided their consent for the use of their radiographic and clinical data. The second group comprised 147 subjects suffering of low back pain from common chronic spinal degenerative disease (column B). None was operated, and none was affected of radicular pain, neurological compression by spinal stenosis or discal compression. They were free of deformities as scoliosis or spondylolysis. Fifty-six subjects described only low back pain (column B1) and ninety-one described an associated leg spreading (column B2).

The third group (column C) comprised 15 children X-rayed for pathologies other than vertebral, aged from 4 to 10 years. Previous studies emphasized sagittal pelvic parameters evolving until 10 years old.

The fourth group comprised 49 spondylolysis cases. Forty of them were with low grade listhesis (LGL) (Meyerling's stage 1 or 2) and without any distortion of the upper sacral plate (column D1). The mean age was of 27 years (SD 12, range 15 to 42 years). The 9 other cases were with high grade listhesis (HGL) (column D2), Meyerling's stage 3 (8 cases) and 4 (1 case). 

The Chairman of our Ethics Committee attested that the data collection of all included patients and healthy subjects was in agreement with the recommendation of the Institutional Review board of the institution.

**Figure 1:**
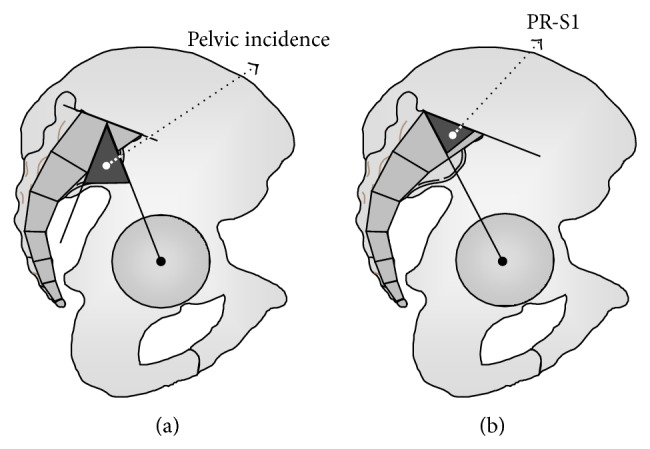
The angular pelvic morphological parameters: the pelvic incidence (PI) and the pelvic lordosis or pelvic radius-S1 angle (PR-S1).

The angular pelvic morphological parameters were (Figure 1) as follows.

- The pelvic incidence (PI): value of the angle between the line perpendicular to the upper plate of the first sacral vertebra (S1) at its midpoint and the line connecting this point to the femoral heads axis.
- The pelvic lordosis or pelvic radius-S1 angle (PR-S1): value of the angle between the sacral upper plate and the line connecting the posterior point of sacral plate to the femoral heads axis.
- Pelvic tilting (PT): value of the angle between the vertical and the line connecting the midpoint of S1 and the femoral heads (parameter here is only used for geometrical demonstration in Appendix B, Figure 5).

Both parameters were proposed for the analysis of the spinopelvic sagittal balance, PI by Duval-Beaupere and colleagues [2, 8], PR-S1 by Jackson [9–11]. 

These angular values were reported in degree. 

**Figure 2:**
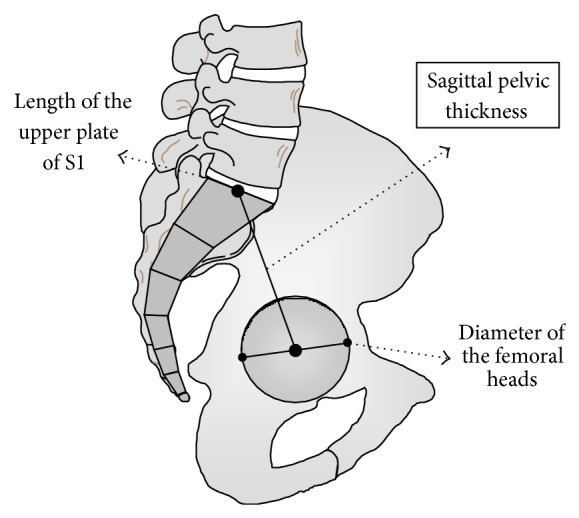
The dimensional parameters: the sagittal pelvic thickness (SPT), the length of S1 and the diameter of the femoral heads.

The dimensional parameters were (Figure 2) as follows.

- The sagittal pelvic thickness (SPT): the distance between the midpoint of the upper plate of S1 and the middle of the femoral heads axis.
- The length of S1: the distance between the anterior and posterior edge of the upper plate of S1.
- The diameter of the femoral heads: mean value of the diameters of two femoral heads.
- The overhang of S1 on the femoral heads (OVS1): distance between the femoral heads and the projection of the midpoint of the upper sacral plate, here expressed relatively to the length of the upper plate of S1 (parameter here is only used for geometrical demonstration in Appendix B, Figure 4).

These values were expressed in millimeter.

Positive angular and dimensional values was defined as posterior, and negative one as anterior.

The values of SPT were also expressed relatively both to the length of the upper plate of S1 and to the diameter of the femoral heads. They were so independent of the height and size of the subjects. Only these relative values were used for the comparisons and the correlations with the angular parameters.

In 4 of the 9 cases of high grade spondylolysis, the upper plate of S1 appeared rounded at its anterior part (really dome shaped sacral plates were not retained because inaccuracy of measurements). Its anterior part was extrapolated from the anterior edge of S1 and the posterior segment of the upper plate.

The student *t*-test was used to investigate the significant differences between the parameters according to the clinical groups. The Spearman's correlation coefficients were reported for the relationships between parameters.

## 3. Results

The mean values and the standard deviations of the parameters were reported in Table 1. The values of PI and PR-S1 observed in our normal population (column A) were similar to the published values assessed as “normal”: 43° to 62° for PI by Duval-Beaupere and colleagues [1, 5], 22° to 42° for PR-S1 by Jackson [2–4]. 

The comparisons between the values of the parameters in each group were reported in Table 2. As previously reported [12], no significant difference was observed between control and low back pain cases. Nevertheless, into the painful group, the “pelvic incidence” was observed significantly lower (and PR-S1 higher) for the cases with leg pain, as well for the SPT expressed according to the length of the upper plate of S1. Conversely, the values of “pelvic incidence” were significantly lower in children (*P* < 0.001) and significantly higher in the spondylolysis groups, mostly for the high grade listhesis. The comparisons were similarly significant for PR-S1.

Obviously, the mean length of the upper plate of S1 was significantly smaller for children and for HGL spondylolysis group than for adults (*P* < 0.001), but similar for adults and LGL spondylolysis group. Also, the values of femoral head diameter were significantly smaller for the spondylolysis groups than for the reference adult normal group (*P* < 0.001), but not according to the listhesis grade (*P* > 0.1). The values of the individual ratios “femoral heads diameter—length of the upper plate of S1” were significantly different between the control group and the others, except with the low grade spondylolysis (*P* > 0.1).

The mean values observed here of femoral head diameter were greater than the published by forensic anthropologists on anatomical specimens [13]. This was connected with the enlargement caused by the diffraction of the R-rays: the calibration device was on the radiographic plate, not at the level of the bony structures. That is why dimensional values were classically considered as dubious (contrarily to angular variables, unaffected by this artefact). It justified expressing the “sagittal pelvic thickness” values relatively to other structures, the femoral heads or the sacral plate.

Expressed in millimeter, the values of the “sagittal pelvic thickness” were significantly greater for the adults than for the children (*P* < 0.001), as well than for spondylolysis subjects with vertebral slip of low grade (*P* < 0.01) and high grade (*P* < 0.05). It was not significantly different between the two spondylolysis groups in spite of different values because of the small number of cases included. On the other hand, expressed relatively to the diameter of the femoral head, the “sagittal pelvic thickness” was observed significantly different only between the children and the adult reference group (*P* < 0.001), and (but lower) the low-grade spondylolysis group (*P* < 0.05). The difference was not observed significant neither between the normal group and the spondylolysis groups nor between the two spondylolysis groups. Nevertheless, expressed relatively to the length of the upper plate of S1, the “sagittal pelvic thickness” was significantly different between the normal adult group and high grade spondylolysis group, and between the two spondylolysis groups. It was similar between adults, children, and low grade spondylolysis groups. 

The Spearman correlation tests between the Pelvic Incidence and the SPT expressed relatively to the femoral head diameter and the upper length of S1 were reported in Table 3. They had a higher significance expressed relatively to the upper plate of S1.

## 4. Discussion

At the same time that the pelvic incidence was described as the key parameter for the analysis of the sagittal balance of the spinopelvic unit, the sagittal pelvic thickness was proposed by Duval-Beaupere and colleagues to define the sagittal anatomy of the pelvis [5]. Boulay et al. showed a significant negative relation (*P* < 0.05) between the “pelvic incidence” and the “sagittal pelvic thickness” (as in our total adult population, *P* < 0.1) [9]. Nevertheless, he observed a better reliability between anatomic and X-ray measurements of the “pelvic incidence” than of the “sagittal pelvic thickness” [9]. It was attributed to the impact of the artifact of radiographic distortion and mostly to the variations of the stature of the subjects on its values. Therefore, we expressed the SPT in proportional value, relating both to the diameter of the femoral heads (as Tardieu et al. [1, 10]) and to the size of the upper plate of S1.

**Figure 3:**
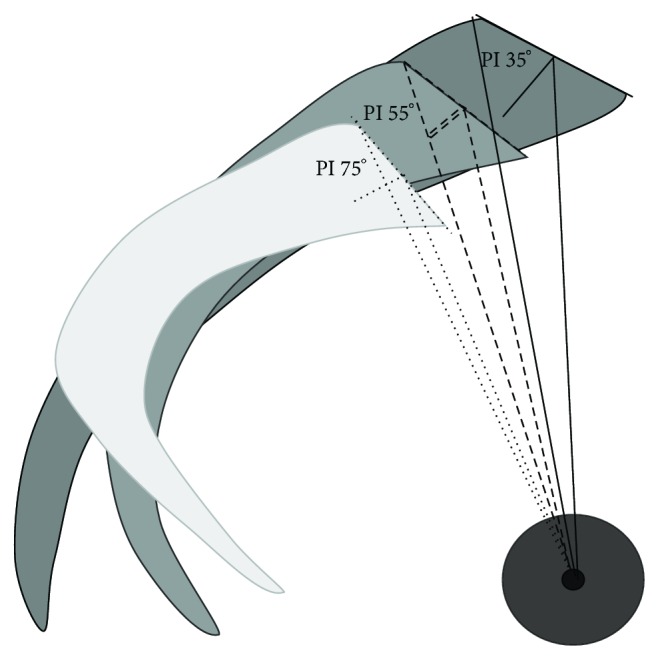
Sacral tilt and sagittal pelvic thickness for low (35°) medium (55°), and high (75°) values of PI.

**Figure 4:**
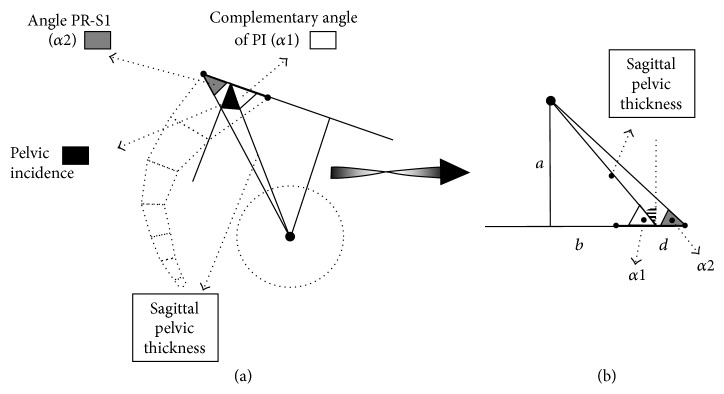
Geometrical connection between sagittal pelvic thickness (SPT), pelvic incidence (PI), and pelvic radius (PR-S1). (a) The angles in a pelvic schema. (b) The angles on rectangular triangles used for the demonstration.

**Figure 5:**
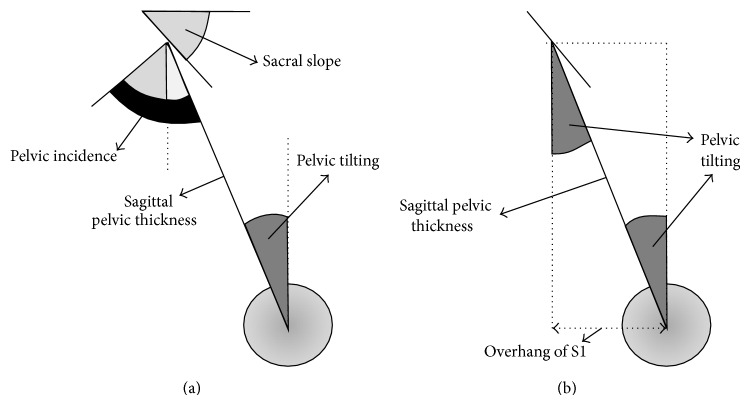
Relations between pelvic incidence (PI), pelvic tilting (PT), overhang of S1 (OVS1), and sagittal pelvic thickness (SPT).

The expression according to the diameter of the femoral heads appeared inappropriate because this diameter was reported not proportional to the stature of the subject and interfering with ethnical factors [11, 13]. On the other hand, the close relationship between “PI” and “PR-S1” (*r* = 0, 998, *P* < 0.001, whatever was the population group) leaded to elaborate a geometrical connection of these two angular parameters with “SPT” (Appendix A). Expressing “SPT” according to the size of the upper plate of S1 was validated. As a result, the correlation's coefficients between “PI” and “SPT/S1” were more significant than with “SPT/TF” (Table 3). So, both sacral tilt and “SPT” were determined by the value of “PI” (Figure 3). The comparisons of the values of such parameters between the population groups also appeared clinically more revealing (Table 2). The value of “SPT/S1” was not observed to be different between the adult subjects, the children, nor the subjects' with spondylolysis of low grade. Conversely, it differed significantly between the spondylolysis case of high grade and both the adult subjects' and the spondylolysis cases of low grade, but not between these spondylolysis cases of high grade and the children. This joined the description of specific features of the sagittal morphology of the sacrum in children and spondylolysis cases [14]. Moreover, sagittal spinopelvic balance was reported of first importance in developmental spondylolysis [15–17]. 

The close relation between “PI” (and “PR-S1”) and “SPT” highlighted the ability of a subject to compensate a sagittal disturbance of the spinopelvic unit. The projection of the gravity of the body segment supported by the pelvic structures (the femoral heads and the sacroiliac joints) was reported to characterize the clinical condition of the sagittal balance of the spinopelvic unit. It was described to be almost vertical and usually posterior to the lumbar segment, the upper plate of S1, and the femoral heads [8, 18, 19] (Appendix B). It was also related to the value of the overhang of S1 on the femoral heads (“OVS1”, expressed in millimetres, longer for high value of “PI”). The impact of “SPT” in this relation was demonstrated in Appendix B. The more or less vertical size of the pelvis was observed influencing the global stability of the spinopelvic unit and its ability to compensate disturbance by more or less efficient sagittal pelvic rotations. Squatter pelvises, with low value of SPT and high value of PI were proved more stable than long vertical pelvises with low value of PI. This was corroborated by the report of a majority of cases with high values of “PI” in soccer and rugby players (sports of power favourable for squatter pelvises) and inversely frequent low values of “PI” in runners (sports of spurting out and endurance) [20].

This more or less ability of the pelvis to react against sagittal disturbance was also connected to a painful torque effect of the gravity on the sacroiliac joints. In upright steady state, the gravity was projecting vertically to the axis of the sacroiliac joints (located at the junction of the first and second sacral vertebra) [8, 18, 19]. No torque was induced to the sacrum, and the posterior rotation of the iliac bones produced by the ground reaction on the femoral heads was counterbalanced by the anterior hip capsule, the ilio-femoral Y ligament of Bigelow, the sacrospinous and sacro-tuberous ligaments, the flexors muscles, and the constrictive under loading wrapped horseshoe form of the acetabulum. This lever arm was longer for high values of “PI”, but acetabular and femoral neck anteversion were reported more pronounced in these cases [1, 10]. Sagittal disruption of this balanced lever was described inducing torque forces in the richly innerved sacroiliac joints. Some postural low back pains were related to sacroiliac torque stresses, sacrospinous and sacrotuberous ligaments strengths, and finally muscular painful contractions (piriformis syndrome [21]). This joints the concept of the “hinge couple” previously suggested in 1983 [22].

As lumbar curvature was reported influencing spinal muscles [23], pelvic morphology was observed affecting the action of pelvic muscles and ligaments. High value of SPT induced both a more vertical lever arm of action and gracilis morphology of these structures. Contrarily, squatter muscles and ligament in “short” pelvises with low value of SPT were more powerful and their lever arm of action more effective because more horizontal. Similarly, the sacrum was reported to be more embedded in cases with a high value of “PI” (and so a low value of “SPT”), so that iliolumbar ligament were shorter, more vertical, and so more effective [24].

## 5. Conclusion

The SPT is a reliable sagittal dimensional anatomical pelvic parameter when it is expressed in value relative to the sizes of the femoral heads. It expresses the capacities of a subject to manage the sources of imbalance at the same time by its spatial compensatory adaptability and the stabilizing aptitudes of the musculoligamentous structures. SPT is strongly connected to the traditional anatomical sagittal angular parameters for the evaluation of the sagittal balance of the spinopelvic unit. Moreover, it allowed a better evaluation of the lever arms of the muscles between pelvis, spine, and hips and the sacroiliac joints. This functional analysis makes it possible to clarify the potential points of actions in revalidation and even avoid useless surgical operations. Clinical treatment should be aimed at improving the stability of the spinopelvic unit taking the sagittal anatomy of the pelvis into account and reducing mechanical stresses.

## Figures and Tables

**Table 1:** 

	A		B		B1		B2		C		D1		D2	
	Normal		Painful cases		Low back pain cases		LBP + leg pain cases		Children		Spondylolysis grade 1-2		Spondylolysis grade 3-4	
	n = 61		n = 147		n = 56		n = 91		n = 15		n = 40		n = 9	
	Men 27	Women 34	Men 69	Women 78	Men 26	Women 30	Men 43	Women 48	Men 6	Women 9	Men 26	Women 14	Men 4	Women 5
	Mean	SD	Mean	SD	Mean	SD	Mean	SD	Mean	SD	Mean	SD	Mean	SD
Age (years)	30.9	7.5	43.1	14.4	42.6	13.7	46.8	17.2	7.2	1.9	27	12	36.5	8.1
Pelvic incidence (PI) (°)	48.3	10.1	49.6	9.8	52.8	9.2	47.3	10.5	40.4	7.8	60.5	11.5	70.6	9.3
Jackson's angle (PR-S1)	36.9	9.1	35.8	8.9	32.5	9.3	38.1	9.9	44	7.7	25.9	10.5	17.2	8.6
Length of the upper plate of S1 (mm)	36	4.2	36	4	37	4.1	36	4	24.5	3.1	34	3.1	28.3	3.8
Mean diameter of the femoral heads (mm)	56.2	6.8	57	7.1	58	7.2	56	6.9	35.4	5.5	52	5.9	48	5.5
Femoral heads diameter/length of S1 (%)	64	5.1	63.2	5.1	63.8	5.2	63.0	6.9	69.2	3	65.3	5.9	53.3	3.4
Sagittal pelvic thickness (SPT) (mm)	132	28.1	132	27.5	130	26.4	134	27.9	84.2	5.8	116	29.5	108.7	24.7
Ratio “SPT/length of S1”	3.33	0.38	3.32	0.31	3.29	0.33	3.41	0.32	3.54	0.32	3.33	0.41	3.79	0.48
Ratio “SPT/femoral heads diameter”	2.12	0.26	2.11	0.26	2.10	0.30	2.16	0.29	2.38	0.31	2.19	0.33	2.29	0.29

Mean values (expressed in degree) and standard deviation of the parameters observed for the entire normal group (column A), the painful cases (column B, B1 only low back pain, B2 with leg spreading), and the children group (column C), the low grade listhesis (column D), and the high grade listhesis spondylolysis group (column E).

**Table 2:** 

	A		B		C		D		E		F		G	
	Normal/painful total		Painful LBP/+leg pain		Normal/ Spondylolysis Grade 1-2		Normal/ Spondylolysis Grade 3-4		Children/ Spondylolysis Grade 1-2		Children/ Spondylolysis Grade 3-4		Spondylolysis Grade 1-2/3-4	
	*t*		*t*		*t*		*t*		*t*		*t*		*t*	
Pelvic incidence (PI)	0.852	NS	**3.333**	**+++**	**5.468**	**+++**	**6.639**	**+++**	**7.408**	**+++**	**8.17**	**+++**	**2.810**	**++**
Jackson's angle (PR-S1)	0.799	NS	**3.459**	**+++**	**5.423**	**+++**	**6.366**	**+++**	**6.9**	**+++**	**7.682**	**+++**	**2.626**	**++**
Length of the upper plate of S1	0.000	NS	1.449	NS	**2.749**	++	**5.596**	**+++**	**10.122**	**+++**	**2.08**	**++**	**4.197**	**+++**
Femoral heads diameter	0.762	NS	1.662	NS	**3.291**	**+++**	**4.040**	**+++**	**9.77**	**+++**	**5.433**	**+++**	1.945	NS
Femoral heads diam./Length S1	1.030	NS	0.798	NS	1.142	NS	**8.180**	**+++**	**3.22**	**++**	**11.583**	**+++**	**8.175**	**+++**
Sagittal pelvic thickness	0.000	NS	0.873	NS	**2.716**	**++**	**2.593**	**++**	**6.491**	**+++**	**2.928**	**++**	0.771	NS
SPT/length of S1	0.182	NS	**2.166**	**+**	0.000	NS	**2.751**	**++**	0.244	NS	0.286	NS	**2.665**	**++**
SPT/femoral heads diameter	0.253	NS	1.193	NS	1.131	NS	1.663	NS	**1.989**	**+**	0.717	NS	0.910	NS

Comparisons of the values of the normal, painful, and spondylolysis groups: between the normal and the total painful group (column A), between the painful cases with and without leg spreading (column B), the normal group and the low grade (column C) and high grade (column D) spondylolysis group, between the children and the low (column E) and high grade spondylolysis group (column F), and between the two spondylolysis groups (column G). + for *P* < 0.05, ++ for *P* < 0.01, +++ for *P* < 0.001.

**Table 3:** 

	Normal		LGL spondylolysis		Children	
	*n* = 61		*n* = 40		*n* = 15	
	*r*	*P*	*r*	*P*	*r*	*P*
SPT/TF	0.237	**<0.05**	0.454	**<0.005**	0.077	N.S.
SPT/S1	0.334	**<0.002**	0.635	**<0.001**	0.3	**<0.2**

Spearman's coefficients of correlation of the relation between pelvic incidence (PI) and the sagittal pelvic thickness (SPT) expressed relating to the femoral heads diameter (SPT/TF) and to the sagittal length of the upper plate of S1 (SPT/S1) for the control, the low grade listhesis spondylolysis, and the children groups.

## Appendices

### A.

Geometrical connection between PI, PR-S1 and SPT (Figure 4).

In Figure 4 (lateral view of the pelvis) the parameters PI and PR-S1 and the requested sagittal  pelvic  thickness, the length between the midpoint of the upper plate of S1, and the bi-coxo-femoral axis were presented. The initially known values usable for the establishment of the geometrical connection were in fat characters. They were as follows.

- The angle “pelvic incidence”: angle between the perpendicular to the upper plate of S1 at its midpoint and the line joining this point to the axis of the femoral heads.
- The angle “*α*1” was the complementary angle of PI: *α*1 = 90° − PI.
- The angle “PR-S1”: angle between the upper plate of S1 and the line joining the posterior edge of the plate of S1 and the axis of the femoral heads. It was named angle “*α*2” for the demonstration.
- The length “*d*” was half of the length of the upper plate of S1: *d*  =  S1/2 (in millimeters).

The sagittal pelvic thickness was wanted: the distance in millimeters between the midpoint of the upper plate of S1 and the axis of the femoral heads.

The perpendicular to the line prolonging the sacral plate was drawing. The distance between the axis of the femoral heads and the intersection of these lines (named point “p”) was named “*a*” (still unknown). The distance between this point and the midpoint of the plate of S1 was named “*b*” (still unknown). “*d*” being known, “*c*” was the distance between the posterior edge of the plate of S1 and the point p (*c* = *b* + *d*).

Figure 4(b) reproduced the same references turned over for a facility of reading.

In this diagram, q1 figured the sought pelvic thickness. It was the hypotenuse of the right-angled triangle whose tops were the midpoint of the plate of S1, the femoral axis and the point p, q2 being the hypotenuse right-angled triangle whose tops were the posterior edge of the plate of S1, the femoral axis and the point p.

Development:

Tangent *α*1 =  *a*/*b* Tangent  *α*2 = *a*/*c*
*b* = *a*/Tangent  *α*1 *a* = *c* · Tangent  *α*2
As *c* = *b* + *d* then *a* = (*b* · Tangent  *α*2) + (*d* · Tangent  *α*2)
As *b* = *a* · Tangent  *α*1, then *a* = (*a* · Tangent  *α*2/Tangent  *α*1) + (*d* · Tangent  *α*2)
*a*− (*a* · Tangent  *α*2/Tangent  *α*1) = (*d* · Tangent  *α*2)
*a* ·  (1 – (Tangent  *α*2/Tangent  *α*1)) = (*d* · Tangent  *α*2)
Consequently “*a*” becomes known:
*a* = (*d* · Tangent  *α*2)/(1 – (Tangent  *α*2/Tangent  *α*1))
*a* being known, *b* becomes known because *b* was observed as *b* = *a*/Tangent  *α*1
The pelvic thickness became consequently calculated as
Spt^2^ = (*a* ^2^ + *b* ^2^)
Spt = square  root  of (*a* ^2^ + *b* ^2^)

### B.

Influence of SPT on the ability of the pelvis to compensate sagittal spinopelvic imbalance.

Data's published is as follows.

1. The projection in the upright position of the gravity of the body segments supported by several structures was investigated [4, 5, 18]. It was described to be almost vertical and usually posterior to the lumbar segment (43 mm (SD 25) behind the midpoint of the inferior plate of L3), the upper plate of S1 (18 mm (SD 27) behind the posterior point), and the femoral heads (36 mm (SD 21) behind on average), directly related with the value of “OVS1”.
2. The centre of gravity of the body segment supported by the pelvis was located at T9 level, 0 to 14 mm before the vertebral body if the thoracic kyphosis was lesser to 35°, from 20 to 32 mm before if the kyphosis was more than 35°.
3. The relationships [18] are as follows:
  SS = 0.5481 · PI + 12.07 (±6.39)
  PI = SS + PT.
4. Development (Figure 5) is as follows:
  PT = PI – SS = 0.4519 · PI − 12.07 (±6.39)
  sin(PT) = OVS1/SPT OVS1 = sin(PT) · SPT.

“SPT” was demonstrated directly related with “PI” and “PRS1” (Appendix A). Consequently, “OVS1” was also affected by the value of “PI” (or “PRS1”). For low value of “PI” (or high value of “PRS1”), the value of “OVS1” is low.

Similarly, the projection of the gravity supported by the femoral heads was more posterior when “PI” value was high (or “PRS1” low). On the other hand, a sagittal rotation of the pelvis will proportionally more influence the value of “OVS1” in the event of low value of “PI” (or high value of “PRS1”), because higher value of “SPT/S1”. So, the squatter pelvises (with high value of “PI”, low value of “SPT/S1, long “OVS1”) were considered less prone to destabilisation than the vertically lengthened pelvises (with low value of “PI”, high value of “SPT/S1, short “OVS1”, easily disturbed by a forward motion of the gravity of the upper body because of short “OVS1”). Inversely, the corrective backwards pelvic rotation appeared less effective when the vertical lever arm of action (“SPT”) was short.

## References

[1] Boulay C., Tardieu C., Bénaim C. (2006). Three-dimensional study of pelvic asymmetry on anatomical specimens and its clinical perspectives. *Journal of Anatomy*.

[2] Jackson R. P., Kanemura T., Kawakami N., Hales C. (2000). Lumbopelvic lordosis and pelvic balance on repeated standing lateral radiographs of adult volunteers and untreated patients with constant low back pain. *Spine*.

[3] Jackson R. P., Peterson M. D., McManus A. C., Hales C. (1998). Compensatory spinopelvic balance over the hip axis and better reliability in measuring lordosis to the pelvic radius on standing lateral radiographs of adult volunteers and patients. *Spine*.

[4] Jackson R. P., Phipps T., Hales C., Surber J. (2003). Pelvic lordosis and alignment in spondylolisthesis. *Spine*.

[5] Legaye J., Duval-Beaupère G., Hecquet J., Marty C. (1998). Pelvic incidence: a fundamental pelvic parameter for three-dimensional regulation of spinal sagittal curves. *European Spine Journal*.

[6] Berge C. (1998). Heterochronic processes in human evolution: an ontogenetic analysis of the hominid pelvis. *American Journal of Physical Anthropology*.

[7] Marks M., Stanford C., Newton P. (2009). Which lateral radiographic positioning technique provides the most reliable and functional representation of a patient's sagittal balance?. *Spine*.

[8] Duval-Beaupere G., Schmidt C., Cosson P. (1992). A barycentremetric study of the sagittal shape of spine and pelvis: the conditions required for an economic standing position. *Annals of Biomedical Engineering*.

[9] Boulay C., Tardieu C., Hecquet J. (2005). Anatomical reliability of two fundamental radiological and clinical pelvic parameters: incidence and thickness. *European Journal of Orthopaedic Surgery and Traumatology*.

[10] Tardieu C., Hecquet J., Boulay C. (2008). Two key describers of the sacro-acetabular relationships: the angles of sacral and acetabular incidence. *Revue de Chirurgie Orthopedique et Reparatrice de l'Appareil Moteur*.

[11] Jantz R. L., Kimmerle E. H., Baraybar J. P. (2008). Sexing and stature estimation criteria for Balkan populations. *Journal of Forensic Sciences*.

[12] Lazennec J.-Y., Ramaré S., Arafati N. (2000). Sagittal alignment in lumbosacral fusion: relations between radiological parameters and pain. *European Spine Journal*.

[13] Giroux C. L., Wescott D. J. (2008). Stature estimation based on dimensions of the bony pelvis and proximal femur. *Journal of Forensic Sciences*.

[14] Marty C., Boisaubert B., Descamps H. (2002). The sagittal anatomy of the sacrum among young adults, infants, and spondylolisthesis patients. *European Spine Journal*.

[15] Hanson D. S., Bridwell K. H., Rhee J. M., Lenke L. G. (2002). Correlation of pelvic incidence with low- and high-grade isthmic spondylolisthesis. *Spine*.

[16] Huang R. P., Bohlman H. H., Thompson G. H., Poe-Kochert C. (2003). Predictive value of pelvic incidence in progression of spondylolisthesis. *Spine*.

[17] Labelle H., Roussouly P., Berthonnaud É., Dimnet J., O'Brien M. (2005). The importance of spino-pelvic balance in L5-S1 developmental spondylolisthesis: a review of pertinent radiologic measurements. *Spine*.

[18] Duval-Beaupere G., Robain G. (1987). Visualization on full spine radiographs of the anatomical connections of the centres of the segmental body mass supported by each vertebra and measured in vivo. *International Orthopaedics*.

[19] Legaye J., Duval-Beaupère G. (2005). Sagittal plane alignment of the spine and gravity a radiological and clinical evaluation. *Acta Orthopaedica Belgica*.

[20] Wodecki P., Guigui P., Hanotel M.-C., Cardinne L., Deburge A. (2002). Sagittal alignment of the spine: comparison between soccer players and subjects without sports activities. *Revue de Chirurgie Orthopedique et Reparatrice de l'Appareil Moteur*.

[21] Kirschner J. S., Foye P. M., Cole J. L. (2009). Piriformis syndrome, diagnosis and treatment. *Muscle and Nerve*.

[22] Vidal J., Marnay T. (1983). A study of antero-posterior trunk balance and pelvic morphology in lumbosacral spondylolisthesis L5 S1. *Revue de Chirurgie Orthopedique et Reparatrice de l'Appareil Moteur*.

[23] McGill S. M., Hughson R. L., Parks K. (2000). Changes in lumbar lordosis modify the role of the extensor muscles. *Clinical Biomechanics*.

[24] Horduna M., Legaye J. (2008). Influence of the sagittal anatomy of the pelvis on the intercrestal line position. *European Journal of Anaesthesiology*.

